# Behavioral and Structural Correlates of Axial Length in School-Aged Children: Baseline Findings from the Seoul Myopia Cohort Study

**DOI:** 10.3390/life16071174

**Published:** 2026-07-16

**Authors:** Ju-Yeun Lee, Chang Hwan Lee, Kyungsik Kim, Un Chul Park, Kunho Bae

**Affiliations:** 1 Department of Ophthalmology, Boston Children’s Hospital, Harvard Medical School, Boston, MA 02115, USA; ju-yeun.lee@childrens.harvard.edu; 2 Department of Preventive Medicine, Seoul National University College of Medicine, Seoul 03080, Republic of Korea; 3 Kim’s Retina Clinic, Cheonan 31127, Republic of Korea; 4 Department of Ophthalmology, Seoul National University Hospital, 101, Daehak-Ro, Jongno-Gu, Seoul 03080, Republic of Korea; 5 Cancer Research Institute, Seoul National University, Seoul 03080, Republic of Korea; 6 Institute of Environmental Medicine, Seoul National University Medical Research Center, Seoul 03080, Republic of Korea; 7 Massachusetts Eye and Ear, Department of Ophthalmology, Harvard Medical School, Boston, MA 02114, USA; 8 Biomedical Research Institute, Seoul National University Hospital, 101, Daehak-Ro, Jongno-Gu, Seoul 03080, Republic of Korea

**Keywords:** pediatric myopia, axial elongation, optical coherence tomography (OCT), smart device usage, viewing distance, Epidemiology

## Abstract

Digital device use has substantially altered children’s visual environments, yet the specific behaviors associated with axial elongation and early retinal remodeling remain unclear in highly urbanized settings. This study investigated biological and behavioral correlates of axial length (AL) and associated macular microstructural changes in preadolescent children. Participants underwent ophthalmic examinations and completed questionnaires assessing lifestyle and digital device use factors. Multivariable linear mixed-effects models identified factors associated with AL and macular thickness. The participant-level prevalence of myopia, defined as spherical equivalent ≤ −0.50 D, was 53.2% and increased from 37.1% in Grade 1 to a peak of 78.7% in Grade 4. Older age, a greater number of myopic parents, flatter corneal curvature, male sex, continuous smart device use exceeding 1 h per session, and viewing distance < 30 cm were independently associated with longer AL. Each 1 mm increase in AL was associated with significant subfoveal choroidal thinning and generalized extrafoveal macular thinning, whereas the central fovea remained relatively preserved. Pediatric AL was associated with hereditary susceptibility, specific digital viewing behaviors, and early extrafoveal retinal structural variation.

## 1. Introduction

The global prevalence of myopia has reached epidemic levels, particularly in highly urbanized regions of East Asia, where it now affects the majority of school-aged children [1]. Pediatric myopia is a major public health concern, as early-onset myopia often progresses to high myopia, substantially increasing the lifetime risk of vision-threatening complications such as myopic maculopathy, retinal detachment, and glaucoma [2,3]. The fundamental pathophysiological driver of these complications is excessive elongation of the globe [4,5]. Identifying modifiable environmental factors underlying this myopic shift is therefore essential for developing effective preventive strategies.

Environmental factors associated with pediatric myopia are multifactorial. Reduced outdoor exposure, intensive educational demands, prolonged near-work activities, and changes in children’s visual environments have all been implicated in the development and progression of myopia [6,7,8,9]. In highly urbanized East Asian settings, these exposures often coexist, making it important to evaluate digital device use within the broader context of lifestyle, educational, and environmental factors rather than in isolation.

In addition to traditional near-work and educational exposures, the rapid adoption of digital smart devices has further altered children’s visual environments, a trend accelerated by the COVID-19 pandemic through increased online learning and indoor confinement [10,11]. As digital media use becomes ubiquitous, conventional metrics such as total “screen time” are insufficient to capture myopiogenic risk [12,13]. Instead, detailed characterization of usage patterns is needed to identify specific behavioral correlates of axial length (AL) during critical periods of ocular development.

Despite this importance, many previous epidemiological studies have been limited by retrospective designs or clinic-based samples, which may be susceptible to selection bias and may not fully capture contemporary environmental exposures in children. Previous pediatric optical coherence tomography (OCT) studies have demonstrated associations between axial length and retinal or macular thickness, providing important evidence that ocular elongation is accompanied by early structural variation [14,15,16,17]. However, a specific knowledge gap remains regarding how these sector-specific spectral domain OCT(SD-OCT)-derived macular and choroidal structural parameters relate to detailed modern visual behavior patterns, such as smart-device viewing distance and continuous session duration, within school-based pediatric populations. Addressing this gap in a highly urbanized setting may help characterize the contemporary phenotype of pediatric axial elongation.

To address these gaps, we established the Seoul Myopia Cohort Study, a prospective school-based cohort designed in a highly urbanized metropolitan setting. Comprehensive on-site examinations including widefield fundus photography, SD-OCT, ocular biometric measurements were conducted alongside comprehensive demographic, familial, perinatal, and detailed lifestyle assessments. This approach minimizes selection bias and enables accurate characterization of real-world environmental exposures and early refractive development.

In this baseline report from the ongoing prospective cohort, we defined axial elongation, assessed using AL, as the primary outcome and evaluated biological, familial, perinatal, educational, outdoor, and digital behavioral factors associated with it. Myopia prevalence based on refractive error and sector-specific macular and choroidal structural parameters measured by SD-OCT were evaluated as secondary outcomes to provide clinical and anatomical context for axial elongation in preadolescent children.

## 2. Materials and Methods

### 2.1. Study Design and Participants

This study is a cross-sectional analysis of baseline data from a school-based prospective cohort, conducted in collaboration with the Seoul Metropolitan Office of Education. Five elementary schools located in Seoul were selected. Eligible participants were students aged 6 to 12 years (grade 1–5) who voluntarily agreed to participate. Children with ocular conditions that could affect refractive status or visual acuity (e.g., intraocular surgery, ocular trauma, or congenital ocular diseases), and those unable to cooperate with ophthalmic examinations or to complete the protocol were excluded. The required sample size was estimated based on previously reported epidemiological models of pediatric myopia. Assuming a conservative prevalence of 50% (1:1 allocation) to maximize the required sample size, a minimum of 326 children was required to detect an odds ratio (OR) of 2.0 for major associated factors with 80% statistical power and a two-sided alpha level of 0.05. To account for an anticipated 10% rate of dropout or incomplete questionnaire responses, the initial recruitment target was set at 363 children. Although axial length was analyzed as the primary continuous outcome in the present baseline report, the original sample size calculation was based on the broader epidemiological objective of characterizing myopia prevalence and associated factors in the cohort.

The study was approved by the Institutional Review Board of Seoul National University Hospital (IRB No. 2312-056-1491; approval date: 26 December 2023). Following IRB approval, an informational letter was distributed to parents of eligible students via the participating schools. Only students who returned written informed consent from a parent or legal guardian were finally enrolled. For children from single-parent households, consent from the primary legal guardian was accepted. This study adheres to the principles of the Declaration of Helsinki.

### 2.2. Baseline Ophthalmologic Examinations

All participants underwent comprehensive ophthalmic examinations conducted by trained examiners. Uncorrected visual acuity (UCVA) and best-corrected visual acuity (BCVA) with current spectacles were measured using a standard 3 m Snellen visual acuity chart, which were subsequently converted to logMAR units. Ocular alignment and the degree of strabismus were evaluated using the prism alternate cover test. Prior to pupillary dilation, ocular biometry, including axial length (AL) and keratometry, was measured using partial coherence interferometry (IOLMaster 500; Carl Zeiss Meditec, Jena, Germany). Macular structural parameters, specifically average macular thickness and total macular volume, were quantitatively evaluated using SD-OCT (Cirrus HD-OCT 5000; Carl Zeiss Meditec, Dublin, CA, USA). Macular thickness was quantified according to the standard Early Treatment Diabetic Retinopathy Study (ETDRS) grid, which divides the macula into nine sectors. Additionally, subfoveal choroidal thickness was assessed using the enhanced depth imaging mode by a masked examiner. Ultra-widefield fundus photography was also performed (Clarus 700; Carl Zeiss Meditec, Dublin, CA, USA).

Following these non-invasive assessments, cycloplegia was induced in participants with both parental and personal informed consent using 1% cyclopentolate. Approximately 40 min after the instillation, cycloplegic autorefraction was performed using an auto-refractometer (ARK-700; Nidek, Gamagori, Japan). Three consecutive measurements were obtained per eye and averaged. The refractive error was expressed as the spherical equivalent (SE), calculated as the spherical power plus one-half of the cylindrical power.

### 2.3. Questionnaire Survey

After completion of baseline ophthalmologic examinations, parents received a link to an online questionnaire through Google Forms (Supplementary Files: Questionnaire_English_Translated and Questionnaire_Original_Korean). The survey consisted of 68 items across six domains: (1) Demographic and Anthropometric factors: age, sex, current height and weight, body mass index (BMI), (2) Family level demographic factors: paternal and maternal age at birth, parental myopia, sibling myopia, birth order, twin status, (3) Perinatal and early-life history factors: maternal diseases (gestational diabetes, thyroid disease, hypertension, maternal smoking during pregnancy), gestational age, birth weight, neonatal oxygen therapy, NICU admission; developmental delay, feeding method, (4) Lifestyle and environmental factors: digital device usage pattern, sleep pattern, outdoor activity time, education/study time, near-work distance, (5) Myopia management history: atropine use/spectacles/orthokeratology, (6) Ocular comorbidities: amblyopia, any type of strabismus. The questionnaire items were adapted from prior epidemiological studies of pediatric myopia and were reviewed and pilot-tested by an expert group consisting of pediatric ophthalmologists, epidemiologists, and representatives from the local education authority. This process was conducted to improve clinical relevance, content appropriateness, clarity, feasibility, and suitability for administration in a school-based setting. Formal psychometric validation and test–retest reproducibility testing in the target study population were not performed.

Within the lifestyle and environmental domain, smart-device use was assessed according to any use, primary device type (smartphone/tablet PC/laptop PC or TV), average daily screen time, maximum daily screen time, average continuous duration per viewing session, viewing frequency, and typical viewing distance. Viewing distance was categorized as <30 cm, 30–50 cm, or ≥50 cm. Time allocation for education and study was assessed by time spent at school, time spent at after-school academies, and homework time. Outdoor activity was assessed by both daily duration and weekly frequency. Sleep patterns were assessed by average sleep duration and habitual bedtime.

### 2.4. Statistical Analysis

Continuous variables are presented as the mean ± standard deviation (SD), and categorical variables are expressed as numbers and percentages. Missing data were handled using complete-case analysis. Refractive error and ocular biometry measurements were available for all included participants. For OCT-derived parameters, the overall missingness rate was 1.45%. Regarding questionnaire data, participants who did not submit the questionnaire were excluded before construction of the final analytical cohort, whereas no partially completed questionnaires with missing individual items were included.

According to the International Myopia Institute (IMI) guidelines, myopia was primarily defined as a spherical equivalent of ≤−0.50 diopters (D) and high myopia was defined as an SE of ≤−6.00 D. The proportion of myopia and high myopia was calculated and stratified by school grade and sex. For descriptive and prevalence analyses, participant-level summaries were generated to avoid treating two eyes from the same participant as independent observations. Myopia and high myopia prevalence were calculated using the worse eye, defined as the eye with the more myopic SE. Participants currently using orthokeratology lenses were excluded from analyses of SE and refractive status because current refraction may not reflect untreated refractive error. AL was summarized using the longer eye. Current myopia management was summarized on a per-participant basis, with participants classified as using spectacles, orthokeratology lenses, or topical atropine if either eye was receiving the corresponding treatment.

The normality of the data distribution was evaluated using the Shapiro–Wilk test prior to analysis. For group comparisons of continuous variables, the independent Student’s t-test or one-way analysis of variance (ANOVA) was used for normally distributed data, whereas the Mann–Whitney U test or Kruskal–Wallis test was applied for non-normally distributed data. Differences in categorical variables were analyzed using the Pearson chi-square test.

To account for the correlation between both eyes within the same participant, linear mixed-effects models (LMMs) were constructed using the lme4 package in R (R Foundation for Statistical Computing, Vienna, Austria). AL was modeled as a continuous outcome variable. In the univariate analysis, each candidate factor including demographic characteristics, family, perinatal, and lifestyle variables was individually assessed for its association with AL. Variables with a *p*-value < 0.05 in the univariate analysis were subsequently included in the multivariate model. In the multivariate LMM, participant ID was specified as a random intercept to account for the nested structure of the data (two eyes per participant).

To quantify the independent contribution of each candidate factor to the variability in AL, variance decomposition analysis was performed based on the multivariate LMM. Coefficient-level partial R^2^ values were calculated from the fixed-effect t statistics in the final multivariable linear mixed-effects model. These values were used to visualize the relative magnitude of each adjusted association with AL. The results were presented as a variable importance plot, in which the length of each bar represents the coefficient-level partial R^2^.

To evaluate macular microstructural changes associated with axial elongation, linear regression models were used to estimate the change in retinal thickness (µm) per 1 mm increase in AL across the nine ETDRS sectors. Additional multivariable models were constructed to assess whether myopia status independently influenced macular thickness after adjusting for AL, thereby isolating the structural effect of axial elongation on macular microstructure. Multivariable models were adjusted for sex, school grade, height, mean keratometry, and central foveal thickness. In analyses of extrafoveal sectors, central foveal thickness was additionally included as a covariate, except when the foveal center was the outcome. To account for multiple comparisons across the nine ETDRS sectors, *p*-values were adjusted using the Benjamini–Hochberg false discovery rate procedure. Formal multiple-testing correction was not applied across all demographic, perinatal, behavioral, and environmental variables evaluated in the AL regression models because these analyses were exploratory.

## 3. Results

### 3.1. Baseline Demographic and Clinical Characteristics

**Figure 1:**
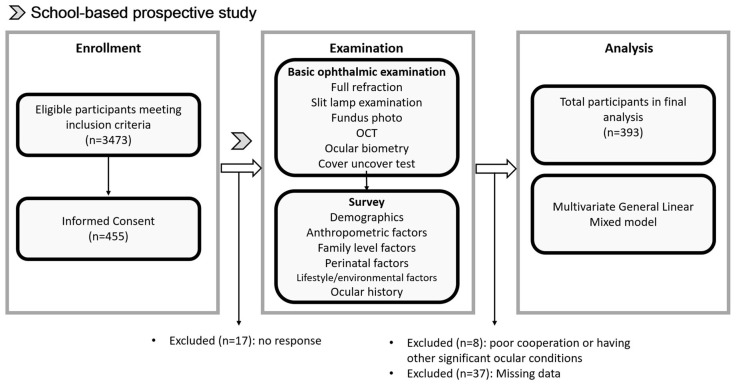
Study flow diagram of participant selection and analytical workflow: Flow diagram illustrating participant selection, data collection procedures, and inclusion in the final multivariate analysis.

A total of 438 Asian participants were recruited from five different elementary schools. Of these, 393 participants (180 boys and 213 girls) who completed the questionnaire (mean grade level of 2.8 ± 1.4) were included in the final analysis (Figure 1). By grade level, there were 93 (23.7%) first-grade students, 92 (23.4%) second-grade students, 79 (20.1%) third-grade students, 73 (18.6%) fourth-grade students, and 56 (14.2%) fifth-grade students (Table 1).

The mean SE of the worse eye was −0.87 ± 1.56 D, and the mean AL of the longer eye was 23.66 ± 0.98 mm. The mean AL increased significantly with advancing school grade, from 23.03 ± 0.87 mm in Grade 1 to 24.28 ± 0.95 mm in Grade 4, followed by a slight decrease to 24.14 ± 1.01 mm in Grade 5 (*p* < 0.001). After excluding participants using orthokeratology lenses from refractive status analyses, the overall participant-level prevalence of myopia was 53.2% (184/346), increasing from 37.1% in Grade 1 to 72.3% in Grade 5, with the highest prevalence observed in Grade 4 (78.7%; *p* < 0.001). High myopia was uncommon overall (1.7%, 6/346) and did not differ significantly across grades. Boys showed a higher proportion of myopia than girls in Grade 1 (39.5% vs. 35.3%). However, the proportion increased more rapidly in girls from Grade 2 onward (50.0% vs. 45.2%), and girls exhibited a substantially higher proportion than boys in Grade 4 (82.8% vs. 75.0%) and Grade 5 (85.7% vs. 61.5%). Current myopia management was also summarized at the participant level; spectacle use, orthokeratology lens use, and topical atropine use were reported in 18.3%, 12.0%, and 4.8% of participants, respectively.

### 3.2. Baseline Patterns of Smart-Device Use and near Work

Among the study participants, 94.9% reported regular use of smart devices, with tablets being the most commonly used device (55.8%), followed by smartphones (35.6%), and laptops or TV (9.2%). Regarding viewing distance, 55.8% of participants used smart devices at a distance of 30–50 cm, 29.6% reported viewing at a distance of <30 cm, and 13.0% at >50 cm. The mean viewing time per session was 1.15 ± 0.91 h (range, 0–4), and the maximum viewing time per session was 1.82 ± 1.35 h (range, 0–8). The average daily viewing time was 1.29 ± 1.25 h (range, 0–10), and participants reported using smart devices on 4.72 ± 2.23 days per week (range, 1–7).

Smart device use patterns differed significantly across grades. Tablet PC use decreased from 75.0% in Grade 1 to 34.6% in Grade 5, while smartphone use increased from 20.2% to 59.6%. Screen exposure also increased with age. Mean daily screen time rose from 1.00 ± 1.41 h in Grade 1 to 1.87 ± 1.59 h in Grade 5 (*p* < 0.001), and maximum session duration increased from 1.43 ± 1.10 to 2.41 ± 1.86 h (*p* < 0.001). Weekly usage frequency increased from 3.97 ± 2.36 to 5.66 ± 2.22 days per week (*p* < 0.001). No significant sex differences were observed in device use (*p* = 0.232) or viewing distance (*p* = 0.495).

### 3.3. Possible Factors Associated with Axial Elongation

Comprehensive results of univariate linear regression analyses of each factor associated with AL are presented in Table S2. In univariate analyses, older age, male sex, and greater anthropometric measures (height and weight) were significantly associated with longer axial length (AL) (all *p* < 0.001). Among familial and perinatal factors, a higher number of myopic parents (*p* = 0.001), greater birth weight (*p* = 0.034), and formula feeding (*p* = 0.037) were significantly associated with longer AL. Regarding environmental and lifestyle factors, longer time spent at school and in after-school academies was associated with increased AL (all *p* < 0.001). For digital device use, primary smartphone use (vs. non-use, *p* = 0.018), shorter viewing distance (*p* = 0.013), and longer average session duration (*p* = 0.009) were significantly associated with longer AL. In contrast, neither the duration nor frequency of outdoor activity was significantly associated with AL (all *p* > 0.05).

**Figure 2:**
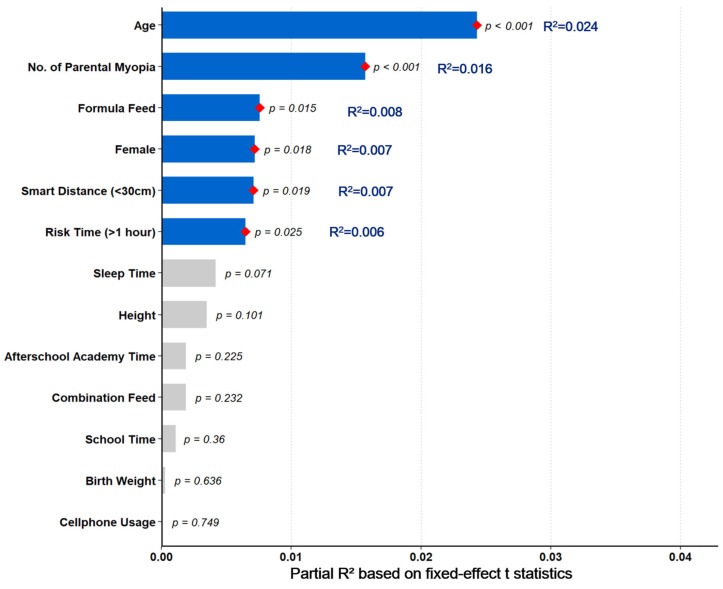
Relative importance of factors associated with axial length. Bar length represents coefficient-level partial R^2^ values calculated from fixed-effect t statistics in the multivariable linear mixed-effects model, with variables ranked by effect size. Blue bars indicate *p* < 0.05, and gray bars indicate *p* ≥ 0.05. Red diamonds denote statistically significant associations. Participant ID was included as a random effect to account for inter-eye correlation.

In a multivariate linear mixed-effects model, several biological and behavioral factors remained independently associated with AL (Table 2). Older age (β = 0.203, *p* < 0.001), a greater number of myopic parents (β = 0.155, *p* < 0.001), flatter corneal curvature (mean keratometry; β = −0.277, *p* < 0.001), and male sex (β = 0.183, *p* = 0.018) were significant biological factors associated with longer AL. Notably, two key digital device–related behaviors remained significant: continuous use exceeding 1 h per session (β = 0.210, *p* = 0.025) and a viewing distance of less than 30 cm (β = 0.195, *p* = 0.019). Additionally, formula feeding was associated with a shorter AL compared with breastfeeding (β = −0.291, *p* = 0.015). In contrast, sleep duration, physical parameters (height and birth weight), primary smart device type, time spent at school, and time spent in after-school academies were not significantly associated with AL. In the variable-importance analysis based on partial R^2^ values, age and number of myopic parents showed the largest relative contributions to AL variation among the evaluated variables, with partial R^2^ values of 0.024 and 0.016, respectively (Figure 2). In contrast, digital viewing behaviors, including smart-device viewing distance < 30 cm and continuous smart-device use exceeding 1 h per session, had smaller partial R^2^ values of 0.007 and 0.006, respectively. These findings indicate that although specific digital viewing behaviors were independently associated with AL, their explanatory contribution was modest compared with established demographic and hereditary factors.

### 3.4. Association Between Axial Length and OCT Microstructure

In multivariable linear mixed-effects models, longer axial length was significantly associated with reduced subfoveal choroidal thickness (β = −25.58, *p* < 0.001), average macular thickness (β = −5.01, *p* < 0.001), and total macular volume (β = −0.18, *p* < 0.001) (Table 3 and Table S3).

**Figure 3:**
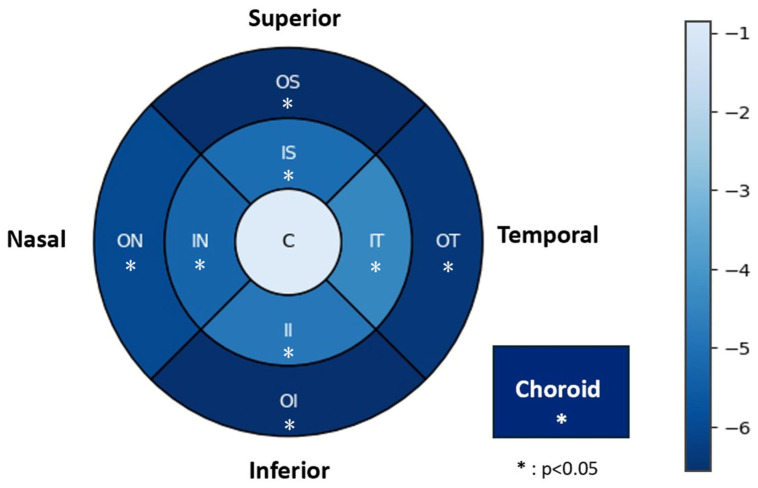
Regional macular structural correlates of axial length. ETDRS-based topographic map illustrating the spatial pattern of associations between macular structure and axial length. Color intensity represents the magnitude and direction of the association.

Topographical analysis revealed generalized thinning across parafoveal and perifoveal regions (all *p* < 0.001), with greater reductions in outer sectors than inner sectors (Figure 3). In contrast, the central fovea showed no significant association with axial length (*p* = 0.350), indicating relative structural preservation.

To distinguish the developmental effect of age from myopic progression, additional multivariable analyses were performed (Table S4). In Model 1 adjusted for AL, older age was associated with increased central foveal thickness (β = 2.24, *p* = 0.025), but not with other macular sectors or choroidal thickness. This pattern was consistent in Model 2 adjusted for SE, where age remained significant only for the central fovea (β = 2.32, *p* = 0.019). These findings suggest that generalized macular and choroidal thinning is primarily driven by axial elongation rather than age.

## 4. Discussion

In this school-based cross-sectional analysis of baseline data from an ongoing prospective cohort of primary school children in Seoul, we examined grade-related patterns in myopia prevalence and axial length and evaluated biological, behavioral, and structural factors associated with AL. Three main findings were observed. First, the prevalence of myopia increased with age and school grades, with a higher prevalence observed among girls in the upper grades. Second, in addition to established demographic and genetic factors, specific digital device usage behaviors, particularly continuous use for more than 1 h and viewing distances of less than 30 cm, were independently associated with axial elongation. Third, axial elongation was associated with a distinct topographic pattern of microstructural remodeling, characterized by parafoveal and perifoveal macular thinning and choroidal thinning, with relative preservation of the central fovea.

This cohort was designed to characterize contemporary patterns of pediatric myopia in a highly urbanized environment. Participant-level myopia prevalence increased substantially across school grades, from 37.1% in Grade 1 to a peak of 78.7% in Grade 4, accompanied by a significant increase in mean AL. A distinct sex-specific pattern was also observed. Boys had longer AL in the multivariable analysis and showed a higher prevalence of myopia in the lower grades, whereas girls demonstrated a steeper grade-related increase in myopia prevalence and a higher prevalence in the upper grades. These findings are consistent with known sex-related differences in ocular dimensions and developmental timing, including larger ocular dimensions in boys and earlier developmental maturation in girls [18,19,20]. Because pubertal status was not directly assessed, the role of pubertal maturation could not be evaluated in this baseline analysis. Longitudinal follow-up incorporating developmental maturation data will be needed to clarify the mechanisms underlying these sex-specific patterns.

When quantifying the independent contributions of associated factors to axial elongation, demographic and familial factors accounted for the greatest explanatory power. Older age, male sex, and a greater number of myopic parents were independently associated with increased AL, supporting the role of hereditary and biologic susceptibility in myopic eye growth. Following these biological determinants, modifiable behavioral factors, particularly patterns of smart device use, were also associated with axial elongation. Rather than total daily screen time, specific usage behaviors, including continuous viewing for more than 1 h and shorter viewing distances of less than 30 cm, were independently associated with longer AL [13,21]. However, the relative explanatory contribution of these digital viewing behaviors was modest compared with major demographic and hereditary factors. Therefore, these findings should not be interpreted as indicating that digital behaviors are dominant contributors to axial elongation. Rather, they suggest that specific viewing behaviors may represent potentially modifiable correlates within a broader multifactorial framework that includes developmental, hereditary, biometric, and environmental factors.

Importantly, because viewing behaviors and AL were assessed at the same baseline visit, the temporal direction of these associations cannot be determined. Reverse causation also cannot be excluded, as children with established myopia or longer AL may be more likely to adopt shorter viewing distances or different patterns of digital device use, potentially because of reduced uncorrected vision. Longitudinal follow-up will therefore be required to determine whether these viewing behaviors precede or follow changes in AL.

In contrast, traditional environmental factors such as outdoor activity were not significantly associated with AL in our cohort. This finding should be interpreted cautiously and should not be considered evidence against the established protective role of outdoor exposure in myopia development. Rather, the lack of association may reflect the highly urbanized and academically competitive context of the study population, in which outdoor exposure may have been relatively limited and homogeneous. In such settings, reduced variability in environmental exposures may make their associations more difficult to detect [2,22]. Consequently, individual susceptibility and specific viewing behaviors, such as very close viewing distance, may appear relatively more prominent in cross-sectional analyses.

Interestingly, our multivariable models indicated that formula feeding was associated with shorter AL than breastfeeding. However, this finding should be interpreted cautiously as exploratory. Although participants were recruited from schools within the same highly urbanized community, which may have reduced heterogeneity in broader neighborhood- and school-level environments, individual-level socioeconomic variables, including household income, parental education, academic achievement, and housing conditions, were not collected. These unmeasured family-level factors may influence both infant feeding practices and early-life myopiogenic exposures, including educational intensity, reading habits, and digital device use [23,24]. Given the limited biological plausibility of a direct effect of infant feeding method on axial elongation, residual confounding by these unmeasured socioeconomic and environmental factors cannot be excluded. Therefore, this association should not be interpreted as evidence of a direct biological effect of feeding method itself on AL.

After examining epidemiological and behavioral correlates of ocular growth, we evaluated OCT-derived structural differences associated with longer AL. A consistent finding was a distinct topographic pattern of retinal remodeling associated with axial elongation. Each 1 mm increase in axial length was independently associated with choroidal thinning and generalized macular thinning across all inner and outer extrafoveal sectors, whereas the central fovea remained relatively preserved without significant thinning.

This spatial gradient is consistent with prior reports suggesting that ocular elongation is accompanied by nonuniform posterior segment structural variation. Because these OCT-derived microstructural differences may accompany physical globe expansion, reliance on refractive measures alone may underestimate concurrent structural alterations, particularly in the presence of optical compensatory mechanisms such as corneal flattening [25]. Although biomechanical stretching has been proposed as one possible explanation for extrafoveal thinning in myopic eyes, the present study did not directly measure biomechanical stress or tissue deformation. Therefore, our findings should be interpreted as cross-sectional structural associations with longer AL rather than direct evidence of a specific mechanical mechanism. The relative preservation of the central fovea may reflect its distinct anatomical features, including a high density of Müller glial cells and the absence of inner retinal layers; however, this interpretation remains hypothetical and requires further longitudinal and mechanistic validation [4,5,17,26].

Furthermore, our analyses of pediatric macular thickness helped distinguish physiological maturation from AL-associated structural variation [16]. After adjustment for AL, older age was independently associated only with increased central foveal thickness, consistent with normal developmental maturation, whereas no independent associations were observed in the extrafoveal sectors or choroid. These findings suggest that central foveal thickening may reflect physiological maturation, whereas extrafoveal macular and choroidal thinning are more closely associated with longer AL. However, because AL and OCT parameters were measured at the same baseline visit, the temporal sequence cannot be determined. Thus, it remains unclear whether these structural differences precede, accompany, or follow axial elongation.

Taken together, these findings indicate that longer AL in preadolescent children is associated with a topographically distinct pattern of macular and choroidal structural differences, characterized by extrafoveal thinning and relative central foveal preservation [16,17,27]. Longitudinal follow-up of this cohort will be necessary to clarify the temporal relationship between axial elongation and these OCT-derived structural differences and to determine whether they predict subsequent myopic progression.

These findings may have clinical relevance by identifying specific viewing patterns that warrant further investigation. Although continuous smart-device use exceeding 1 h and viewing distances below 30 cm were associated with longer AL, longitudinal confirmation is needed before these thresholds can be translated into formal clinical recommendations. Nevertheless, these findings may inform individualized counseling on near-work behaviors and support closer monitoring in children with potentially high-risk viewing patterns.

Several limitations of this study should be acknowledged. First, although derived from an ongoing prospective cohort, the present analysis is cross-sectional and based on baseline data. Therefore, temporal relationships among lifestyle factors, axial elongation, and retinal structural changes cannot be established, and the observed associations should not be interpreted as evidence of causality. Reverse causation also cannot be excluded, as children with established myopia or longer AL may adopt shorter viewing distances or different patterns of digital device use. It also remains unclear whether the observed macular and choroidal structural differences precede, accompany, or follow axial elongation. Although the topographic pattern of retinal and choroidal thinning is consistent with proposed biomechanical models of axial elongation, biomechanical changes were not directly measured and therefore cannot be established as the underlying mechanism. Longitudinal follow-up is needed to clarify these temporal and mechanistic relationships. Second, potential selection bias or a cohort effect may have been present. Because participation was voluntary, families with greater interest in eye health or greater willingness to complete the study procedures may have been more likely to enroll. In addition, the slight reduction in mean AL in the fifth grade suggests that older students with more severe myopia may have been underrepresented. Third, the study population was drawn from a highly urbanized district in Seoul, which may limit generalizability, particularly with respect to environmental exposures. The lack of a rural comparison group also limited evaluation of environmental variability, including the potential protective effects of outdoor activity. Fourth, parental socioeconomic status, household income, and parental education were not assessed. This limitation restricts our ability to fully account for residual confounding, particularly in interpreting the association between feeding methods and AL, as socioeconomic and educational factors may influence both infant feeding practices and early-life environmental exposures. Fifth, visual acuity was measured using a standard Snellen chart and converted to logMAR values for descriptive statistical reporting. Although this approach was practical for on-site school-based screening of young children, it is less standardized and less precise than formal logMAR-based chart testing, particularly at lower acuity levels. Finally, lifestyle behaviors were assessed using parent-assisted questionnaires and may be subject to recall bias and social desirability bias. Although the questionnaire was adapted from prior epidemiological studies and underwent expert review and pilot testing, formal psychometric validation and test–retest reproducibility testing were not performed in the target study population. In particular, smart-device exposure, viewing distance, outdoor activity, educational workload, and sleep patterns were based on parental report rather than objective monitoring. In addition, because formal multiple-testing correction was not applied across all questionnaire-derived and demographic variables in these exploratory analyses, some associations should be interpreted cautiously.

In conclusion, this school-based baseline analysis showed that longer AL in children from a highly urbanized environment was associated with hereditary susceptibility, sex-specific developmental patterns, and specific digital viewing behaviors. Demographic and hereditary factors accounted for the greatest explanatory contribution, followed by specific digital viewing behaviors, including shorter viewing distance and prolonged continuous use. Longer AL was also associated with structural differences, particularly outer macular thinning. These findings suggest that modifiable behavioral factors may inform future longitudinal studies and individualized counseling strategies for children at risk of myopia progression.

## Figures and Tables

**Table 1:** Baseline Demographic, Clinical, and Behavioral Characteristics of the Study Population by School Grade.

Characteristics	Total (*N* = 393)	Grade 1 (*N* = 93)	Grade 2 (*N* = 92)	Grade 3 (*N* = 79)	Grade 4 (*N* = 73)	Grade 5 (*N* = 56)	*p* Value ^†^
**Sex,** ***n*** **(%)**							0.362
Boys	180 (45.8)	40 (43.0)	43 (46.7)	30 (38.0)	37 (50.7)	30 (53.6)	
Girls	213 (54.2)	53 (57.0)	49 (53.3)	49 (62.0)	36 (49.3)	26 (46.4)	
**Ocular Biometry** ^‡^							
Spherical equivalent, D	−0.87 ± 1.56	−0.34 ± 1.29	−0.62 ± 1.17	−0.75 ± 1.45	−1.70 ± 1.89	−1.43 ± 1.73	<0.001
Axial length, mm	23.66 ± 0.98	23.03 ± 0.87	23.44 ± 0.75	23.74 ± 0.83	24.28 ± 0.95	24.14 ± 1.01	<0.001
**Proportion of Myopia** ^‡^**,** ***n*** **(%)**							
Overall	184/346 (53.2)	33/89 (37.1)	40/84 (47.6)	29/65 (44.6)	48/61 (78.7)	34/47 (72.3)	<0.001
Boys	88/168 (52.4)	15/38 (39.5)	21/42 (50.0)	12/30 (40.0)	24/32 (75.0)	16/26 (61.5)	0.018
Girls	96/178 (53.9)	18/51 (35.3)	19/42 (45.2)	17/35 (48.6)	24/29 (82.8)	18/21 (85.7)	<0.001
**High Myopia** ^‡^**,** ***n*** **(%)**	6/346 (1.7)	0/89 (0.0)	1/84 (1.2)	0/65 (0.0)	3/61 (4.9)	2/47 (4.3)	0.083
**Current Myopia Management** ^‡^**,** ***n*** **(%)**							
Spectacles (Glasses)	72 (18.3)	10 (10.8)	7 (7.6)	13 (16.5)	25 (34.2)	17 (30.4)	<0.001
Orthokeratology (Dream lens)	47 (12.0)	4 (4.3)	8 (8.7)	14 (17.7)	12 (16.4)	9 (16.1)	0.028
Topical Atropine	19 (4.8)	4 (4.3)	7 (7.6)	5 (6.3)	2 (2.7)	1 (1.8)	0.433
**Daily Time on Activities (hours/day)**							
Afterschool academy	2.26 ± 1.30	1.98 ± 1.38	2.22 ± 1.33	2.31 ± 1.16	2.57 ± 1.27	2.36 ± 1.31	0.061
Homework	1.45 ± 0.81	1.33 ± 0.96	1.43 ± 0.69	1.39 ± 0.66	1.53 ± 0.71	1.68 ± 0.99	0.103
Outside activities	1.90 ± 1.39	1.80 ± 1.05	1.75 ± 1.30	1.81 ± 1.30	2.05 ± 1.63	2.26 ± 1.74	0.169
Outside frequency	3.20 ± 2.36	3.43 ± 2.31	3.07 ± 2.02	2.92 ± 2.02	3.29 ± 2.76	3.34 ± 2.84	0.637
**Digital smart device use,** ***n*** **(%)**							0.191
Current users	373 (94.9)	85 (91.4)	87 (94.6)	78 (98.7)	71 (97.3)	52 (92.9)	
**Primary device type,** ***n*** **(%)**							<0.001
Smartphone	132 (35.6)	17 (20.2)	24 (27.6)	28 (35.9)	32 (45.7)	31 (59.6)	
Tablet PC	205 (55.3)	63 (75.0)	49 (56.3)	43 (55.1)	32 (45.7)	18 (34.6)	
TV/Laptop/Others	34 (9.2)	4 (4.8)	14 (16.1)	7 (9.0)	6 (8.6)	3 (5.8)	
**Viewing distance,** ***n*** **(%)**							0.167
<30 cm	115 (29.6)	19 (20.9)	27 (29.7)	27 (34.6)	21 (28.8)	21 (37.5)	
30–50 cm	206 (55.8)	54 (65.1)	45 (52.3)	37 (48.1)	41 (57.7)	29 (55.8)	
>50 cm	48 (13.0)	10 (12.0)	14 (16.3)	13 (16.9)	9 (12.7)	2 (3.8)	
**Viewing duration**							
Average daily screen time (hours/day)	1.29 ± 1.25	1.00 ± 1.41	1.10 ± 0.94	1.16 ± 0.82	1.58 ± 1.27	1.87 ± 1.59	<0.001
Maximum daily screen time (hours/day)	1.82 ± 1.35	1.43 ± 1.10	1.56 ± 1.02	1.72 ± 1.12	2.30 ± 1.51	2.41 ± 1.86	<0.001
Average duration per session (hours/session)	1.15 ± 0.91	0.83 ± 0.52	1.08 ± 0.80	1.22 ± 0.98	1.29 ± 0.81	1.48 ± 1.36	<0.001
Weekly viewing frequency (days/week)	4.72 ± 2.33	3.97 ± 2.36	4.36 ± 2.45	4.95 ± 2.05	5.14 ± 2.18	5.66 ± 2.22	<0.001

Data are expressed as mean (standard deviation) for continuous variables and as number (percentage) for categorical variables. ^†^ *p*-values were calculated using the Kruskal–Wallis test for continuous variables. For categorical variables, Fisher’s exact test with Monte Carlo simulation (10,000 replicates) was utilized to account for small expected frequencies. ^‡^ Ocular biometric parameters, myopia prevalence, high myopia prevalence, and current myopia management were summarized on a per-participant basis. Spherical equivalent and refractive status were based on the worse eye, defined as the eye with the more myopic spherical equivalent. Axial length was summarized using the longer eye. Participants using orthokeratology lenses were excluded from refractive status and myopia prevalence analyses.

**Table 2:** Associations between possible factors and axial length across various linear mixed models.

Variable	Univariate			Multivariate		
	Beta	95% CI	*p* Value	Beta	95% CI	*p* Value
Age	0.278	(0.218, 0.338)	<0.001	0.203	(0.113, 0.294)	<0.001
Sex	0.415	(0.228, 0.602)	<0.001	0.183	(0.032, 0.334)	0.018
Number of myopic parents	0.186	(0.074, 0.297)	0.001	0.155	(0.068, 0.242)	<0.001
Height	0.043	(0.034, 0.051)	<0.001	0.011	(−0.002, 0.024)	0.101
Birth weight	0.215	(0.017, 0.413)	0.034	−0.039	(−0.198, 0.121)	0.636
Formula feeding	−0.315	(−0.611, −0.019)	0.037	−0.291	(−0.526, −0.056)	0.015
Mean keratometry	−0.294	(−0.337, −0.250)	<0.001	−0.277	(−0.318, −0.236)	<0.001
Sleep duration	−0.177	(−0.309, −0.046)	0.008	0.104	(−0.009, 0.218)	0.071
School time	0.257	(0.146, 0.369)	<0.001	0.044	(−0.050, 0.138)	0.360
After school academy time	0.082	(0.009, 0.155)	0.028	0.035	(−0.022, 0.093)	0.225
Smartphone usage	0.544	(0.093, 0.996)	0.018	−0.026	(−0.187, 0.135)	0.749
Viewing distance < 30 cm	0.263	(0.054, 0.472)	0.014	0.195	(0.032, 0.358)	0.019
Average session duration > 1 h	0.332	(0.100, 0.563)	0.005	0.210	(0.027, 0.393)	0.025

**Table 3:** Univariate and multivariate associations between macular optical coherence tomography (OCT) parameters and axial length.

OCT Parameters	Univariate Estimate (95% CI)	*p* Value	Multivariate Estimate (95% CI)	Standardized β	*p* Value ^†^
Macular Subfield Thickness, μm					
Fovea center	1.23 (−0.29, 2.75)	0.114	−0.88 (−2.72, 0.96)	−0.049	0.350
Inner superior	−2.88 (−4.20, −1.57)	<0.001	−5.10 (−6.68, −3.52)	−0.335	<0.001
Inner temporal	−2.41 (−3.71, −1.11)	<0.001	−4.44 (−5.99, −2.88)	−0.294	<0.001
Inner inferior	−3.00 (−4.31, −1.68)	<0.001	−4.80 (−6.41, −3.18)	−0.318	<0.001
Inner nasal	−3.50 (−4.83, −2.17)	<0.001	−5.30 (−6.89, −3.70)	−0.340	<0.001
Outer superior	−5.61 (−6.76, −4.47)	<0.001	−6.64 (−8.08, −5.19)	−0.476	<0.001
Outer temporal	−4.78 (−5.87, −3.69)	<0.001	−6.43 (−7.76, −5.09)	−0.484	<0.001
Outer inferior	−5.88 (−7.01, −4.75)	<0.001	−6.60 (−8.05, −5.16)	−0.469	<0.001
Outer nasal	−5.05 (−6.30, −3.80)	<0.001	−5.99 (−7.55, −4.43)	−0.396	<0.001
Subfoveal choroidal thickness	−18.93 (−22.65, −15.20)	<0.001	−25.58 (−30.29, −20.88)	−0.564	<0.001
Global Parameters					
Average thickness, μm	−4.21 (−5.25, −3.16)	<0.001	−5.01 (−6.30, −3.71)	−0.397	<0.001
Macular volume, mm^3^	−0.15 (−0.19, −0.12)	<0.001	−0.18 (−0.23, −0.14)	−0.396	<0.001

*Abbreviations*: CI, confidence interval. ^†^ *p*-values were adjusted for multiple comparisons across the topographic sectors using the Benjamini–Hochberg false discovery rate method. The ETDRS macular grid was defined as follows: the central foveal subfield corresponded to the central 1 mm circle, the inner sectors to the 1 to 3 mm ring, and the outer sectors to the 3 to 6 mm ring.

## Data Availability

The data presented in this study are available on request from the corresponding author.

## Supplementary Materials

The following supporting information can be downloaded at: https://www.mdpi.com/article/10.3390/life16071174/s1, Table S1: Unadjusted associations between candidate factors and axial length in the study population; Table S2: Absolute macular OCT parameters according to grade; Table S3: Multivariable associations of chronological age with optical coherence tomography parameters, adjusted for axial length (AL, Model 1) or spherical equivalent (SE, Model 2); Questionnaire S1: Complete English translation of the parent-assisted myopia environmental factors questionnaire; Questionnaire S2: Original Korean version of the parent-assisted myopia environmental factors questionnaire.
