# Supplementary material for: Behavioral and Structural Correlates of Axial Length in School-Aged Children: Baseline Findings from the Seoul Myopia Cohort Study
Source: Life (Basel). 2026 Jul 16;16(7):1174. doi: 10.3390/life16071174 (PMC13412910; doi:10.3390/life16071174)
Supplement: Supplementary file 1 [file life-16-01174-s001.zip › Questionnaire_English_Translated.pdf]

Myopia Environmental Factors Questionnaire

Complete English Translation of the Original Korean Google Forms Questionnaire

Purpose and instructions shown in the original form

Myopia often begins around 5-10 years of age and tends to stop progressing around age 20; however, the progression rate is particularly rapid among elementary-school children. Through this questionnaire, we aim to evaluate family history and environmental factors related to myopia, including eyeglass or lens use, and to identify risk factors for progression to high myopia. Parents who complete this questionnaire carefully will receive detailed examination results related to their child's eye health; therefore, we ask for your participation.
- Estimated completion time: approximately 5 minutes.
- Because many questions require answers from the mother, we ask that the child's mother complete the questionnaire whenever possible.
- Questionnaire results will never be publicly disclosed. They will be used only for research purposes and destroyed after completion of the study.
- In the original Google Form, an asterisk (*) indicated a required question.

Clarification note for the translated supplementary file

This document is an English translation/clarification of the original Korean Google Forms questionnaire. Some section titles in the original form were unnamed; section labels and branching descriptions in this file were added only to clarify the respondent flow. Asterisks (*) indicate required questions in the original Google Form. Conditional items were required only when displayed to eligible respondents. If a response was missing or outside the appropriate range, the Google Form was configured to prevent respondents from proceeding to the next section or completing the survey.

Main branching/skip logic

- Q12: If the child was a twin, the respondent answered Q46 and then continued to Q13.
- Q15: If the mother smoked before or during pregnancy, the respondent answered Q47-Q49 and then returned to Q16.
- Q16: If the mother drank alcohol during pregnancy, the respondent answered Q50-Q51 and then returned to Q17.
- Q37: If the child wore eyeglasses at least once per week, the respondent answered Q52-Q55 and then returned to Q38.
- Q38: If the child wore orthokeratology/night lenses at least once per week, the respondent answered Q56-Q60 and then returned to Q39.
- Q39: If the child used atropine eye drops at least once per week, the respondent answered Q61-Q64 and then returned to Q40.
- Q45: If the child used or watched a smart device at least once per week, the respondent answered Q65-Q70. If not, the questionnaire ended after Q45.

Complete English translation of questions and response choices

No.	Required in original form	English translation of item shown to respondent	Response format and choices	Display condition / branching
1	Yes	Email address *	Open text field.	Displayed to all respondents.
2	Yes	What is the child's name? *	Open text field.	Displayed to all respondents.
3	Yes	Please enter the child's current grade, class, and student number. For example, if the child is in grade 3, class 4, number 12, enter 030412. *	Open text field.	Displayed to all respondents.
4	Yes	What is the child's date of birth? *	Date field. Example shown in the original form: January 7, 2019.	Displayed to all respondents.
5	Yes	What is the child's most recently measured height? Enter numbers only, in centimeters. *	Numeric field.	Displayed to all respondents.
6	Yes	What is the child's most recently measured weight? Enter numbers only, in kilograms. *	Numeric field.	Displayed to all respondents.
7	Yes	What is the name of the school the child attends? *	Single choice:<br>- Jamil Elementary School<br>- Jamsin Elementary School	Displayed to all respondents.

No.	Required in original form	English translation of item shown to respondent	Response format and choices	Display condition / branching
			- Sincheon Elementary School<br>- Jamjeon Elementary School<br>- Seokchon Elementary School	
8	Yes	What is the child's sex? *	Single choice:<br>- Male<br>- Female	Displayed to all respondents.
9	Yes	At how many gestational weeks was the child born? A typical full-term pregnancy is 40 weeks. Enter numbers only, in weeks. *	Numeric field.	Displayed to all respondents.
10	Yes	What was the child's birth weight? If possible, enter to one decimal place; numbers only. *	Numeric field.	Displayed to all respondents.
11	Yes	What is the child's birth order? *	Single choice:<br>- First child<br>- Second child<br>- Third or later child<br>- Only child	Displayed to all respondents.
12	Yes	Is the child a twin? *	Single choice:<br>- Yes<br>- No	If Yes: go to Q46, then continue to Q13.<br>If No: continue to Q13.
13	Yes	How old was the father when the child was born? Enter completed age in years; numbers only. *	Numeric field.	Displayed after Q12/Q46 branching.
14	Yes	How old was the mother when the child was born? Enter completed age in years; numbers only. *	Numeric field.	Displayed to eligible respondents after the preceding section.
15	Yes	For the mother: Did you smoke before pregnancy or during pregnancy? *	Single choice:<br>- No<br>- Yes	If Yes: answer Q47-Q49, then return to Q16.<br>If No: continue to Q16.
16	Yes	For the mother: Did you drink alcohol during pregnancy? *	Single choice:<br>- No<br>- Yes	If Yes: answer Q50-Q51, then return to Q17.<br>If No: continue to Q17.
17	Yes	For the mother: Were you ever diagnosed with hypertension during pregnancy, including preeclampsia/pregnancy-induced hypertension? *	Single choice:<br>- No<br>- Yes	Displayed to all eligible respondents.
18	Yes	For the mother: Were you ever diagnosed with diabetes during pregnancy? *	Single choice:<br>- No<br>- Yes	Displayed to all eligible respondents.
19	Yes	For the mother: Were you diagnosed with any other disease during pregnancy besides the conditions listed above? If yes, please describe it in the Other field. *	Single choice:<br>- No<br>- Other: [free text]	Displayed to all eligible respondents.
20	Yes	What was the child's main feeding method during the first 3 months after birth? *	Single choice:<br>- Breastfeeding<br>- Formula feeding<br>- Mixed feeding (breast milk and formula)<br>- Other: [free text]	Displayed to all respondents.
21	Yes	Has the child ever received treatment in a neonatal intensive care unit (NICU) after birth? *	Single choice:<br>- No<br>- Yes	Displayed to all respondents.
22	Yes	Has the child ever been diagnosed with a respiratory disease or received oxygen treatment during the fetal/neonatal period? *	Single choice:<br>- No<br>- Yes<br>- Do not know	Displayed to all respondents.
23	Yes	Has the child had a regular ophthalmologic examination or vision screening within the past year? *	Single choice:<br>- No<br>- Yes	Displayed to all respondents.
24	Yes	Has the child ever been diagnosed with or treated for retinopathy of	Single choice:	Displayed to all respondents.

No.	Required in original form	English translation of item shown to respondent	Response format and choices	Display condition / branching
		prematurity? *	- No<br>- Yes	
25	Yes	Has the child ever been diagnosed with amblyopia? *	Single choice:<br>- No<br>- Yes	Displayed to all respondents.
26	Yes	Has the child ever been diagnosed with strabismus? *	Single choice:<br>- No<br>- Yes	Displayed to all respondents.
27	Yes	Has the child ever been diagnosed with any other congenital eye disease? If yes, please describe it in the Other field. *	Single choice:<br>- No<br>- Do not know<br>- Other: [free text]	Displayed to all respondents.
28	Yes	Has the child ever visited a developmental clinic because of delayed speech or behavioral development? *	Single choice:<br>- No<br>- Yes	Displayed to all respondents.
29	Yes	Has the child ever been diagnosed with a developmental disorder? *	Single choice:<br>- No<br>- Yes	Displayed to all respondents.
30	Yes	Is the child currently taking a multivitamin, including vitamin D? *	Single choice:<br>- No<br>- Yes<br>- Do not know	Displayed to all respondents.
31	Yes	During the past 2 weeks, what was the child's average sleep duration? Enter numbers only, in hours. *	Numeric field.	Displayed to all respondents.
32	Yes	During the past 2 weeks, what was the child's average bedtime? Use 24-hour time and enter the rounded hour only; for example, enter 23 for 11:00 PM. *	Numeric field.	Displayed to all respondents.
33	Yes	Please indicate the child's experience with growth hormone treatment. *	Single choice:<br>- Currently receiving continuous growth hormone treatment<br>- Previously received growth hormone treatment but not currently<br>- No experience	Displayed to all respondents.
34	Yes	Please indicate whether either parent has myopia. If a parent has had refractive surgery such as LASIK, answer based on the refractive status before surgery. *	Single choice:<br>- Father<br>- Mother<br>- Both parents have myopia<br>- Neither parent has myopia	Displayed to all respondents.
35	Yes	Please indicate all siblings of the child who have myopia. If a sibling has had refractive surgery, answer based on the refractive status before surgery. *	Select all that apply:<br>- Not applicable, only child<br>- Older brother<br>- Younger brother<br>- Older sister<br>- Younger sister<br>- Has siblings but none have myopia	Displayed to all respondents.
36	Yes	To your knowledge, does the child currently have myopia? *	Single choice:<br>- No<br>- Yes	Displayed to all respondents.
37	Yes	Does the child currently wear eyeglasses at least once per week? *	Single choice:<br>- No<br>- Yes	If Yes: answer Q52-Q55, then return to Q38.<br>If No: continue to Q38.
38	Yes	Does the child currently wear orthokeratology lenses/night lenses (Dream Lens) at least once per week? *	Single choice:<br>- No	If Yes: answer Q56-Q60, then return to Q39.

No.	Required in original form	English translation of item shown to respondent	Response format and choices	Display condition / branching
			- Yes	If No: continue to Q39.
39	Yes	Does the child currently use atropine eye drops at least once per week? *	Single choice:<br>- No<br>- Yes	If Yes: answer Q61-Q64, then return to Q40.<br>If No: continue to Q40.
40	Yes	What is the child's average daily school class time? Exclude school vacations; enter average hours per day. *	Numeric field.	Displayed to all respondents after the myopia-treatment branching section.
41	Yes	What is the child's average daily private academy/tutoring class time, including private learning academies and workbook/home-study programs? Include school vacations; enter average hours per day. *	Numeric field.	Displayed to all respondents.
42	Yes	What is the child's average daily reading/homework time? Include school vacations; enter average hours per day. *	Numeric field.	Displayed to all respondents.
43	Yes	What is the child's average daily outdoor activity time? Include weekends; enter numbers only, in hours. Outdoor activity refers to all activities performed outdoors, including commuting to and from school. *	Numeric field.	Displayed to all respondents.
44	Yes	On average, how many times per month does the child participate in nature-related activities such as walks, picnics, camping, or forest activities? Enter the average number of days per month. *	Numeric field.	Displayed to all respondents.
45	Yes	Does the child use or watch a smart device (tablet, smartphone, or laptop) at least once per week? *	Single choice:<br>- No<br>- Yes	If Yes: answer Q65-Q70.<br>If No: the questionnaire ends after Q45.
46	Conditional	If the child is a twin: Is the child an identical or fraternal twin?	Single choice:<br>- Identical twin<br>- Fraternal twin	Displayed only if Q12 = Yes. After answering, continue to Q13.
47	Conditional	For the mother: Before pregnancy, how many cigarettes did you smoke per day on average? Enter average cigarettes per day.	Numeric field.	Displayed only if Q15 = Yes.
48	Conditional	For the mother: Before pregnancy, what was your total duration of smoking? Round to the nearest year.	Numeric field.	Displayed only if Q15 = Yes.
49	Conditional	For the mother: When did you quit smoking?	Single choice:<br>- Before pregnancy<br>- Immediately after pregnancy began to gestational week 12<br>- Gestational weeks 13-46<br>- After gestational week 46<br>- Did not quit	Displayed only if Q15 = Yes. After answering, return to Q16.
50	Conditional	For the mother: During pregnancy, how often did you drink alcohol on average? Enter number of drinking occasions per week.	Numeric field.	Displayed only if Q16 = Yes.
51	Conditional	For the mother: During pregnancy, how much alcohol did you drink on average? Enter the average number of glasses per drinking occasion, regardless of alcohol type. Example: 1 bottle of soju = 8 glasses; 1 bottle of beer = 3 glasses.	Numeric field.	Displayed only if Q16 = Yes. After answering, return to Q17.
52	Conditional	At what age did the child start wearing eyeglasses? Enter completed age in years.	Numeric field.	Displayed only if Q37 = Yes.
53	Conditional	During the past 2 weeks, how many hours per day did the child wear eyeglasses on average? Enter numbers only, in hours.	Numeric field.	Displayed only if Q37 = Yes.
54	Conditional	On average, how many days per week does the child wear eyeglasses?	Numeric field.	Displayed only if Q37 = Yes.
55	Conditional	How long has it been since the child was prescribed the currently worn eyeglasses? Enter numbers only, in months.	Numeric field.	Displayed only if Q37 = Yes. After answering, return to Q38.
56	Conditional	At what age did the child start wearing orthokeratology lenses/night lenses (Dream Lens)? Enter completed age in years.	Numeric field.	Displayed only if Q38 = Yes.
57	Conditional	Which eye(s) does the child wear orthokeratology lenses/night lenses in?	Single choice:<br>- Both eyes<br>- Right eye only<br>- Left eye only	Displayed only if Q38 = Yes.

No.	Required in original form	English translation of item shown to respondent	Response format and choices	Display condition / branching
58	Conditional	During the past 2 weeks, how many hours per day did the child wear orthokeratology lenses/night lenses on average? Enter numbers only, in hours.	Numeric field.	Displayed only if Q38 = Yes.
59	Conditional	On average, how many days per week does the child wear orthokeratology lenses/night lenses?	Numeric field.	Displayed only if Q38 = Yes.
60	Conditional	How long has it been since the child was prescribed the currently worn orthokeratology lenses/night lenses? Enter numbers only, in months.	Numeric field.	Displayed only if Q38 = Yes. After answering, return to Q39.
61	Conditional	At what age did the child start using atropine eye drops? Enter completed age in years.	Numeric field.	Displayed only if Q39 = Yes.
62	Conditional	In which eye(s) does the child use atropine eye drops?	Single choice:<br>- Both eyes<br>- Right eye<br>- Left eye	Displayed only if Q39 = Yes.
63	Conditional	What is the concentration of atropine currently being used?	Single choice:<br>- 0.1%<br>- 0.05%<br>- 0.025%<br>- 0.01%<br>- Do not know	Displayed only if Q39 = Yes.
64	Conditional	On average, how many days per week does the child use atropine eye drops?	Numeric field.	Displayed only if Q39 = Yes. After answering, return to Q40.
65	Conditional	During the past 2 weeks, which smart device did the child use most frequently?	Single choice:<br>- Tablet (e.g., iPad, Galaxy Tab)<br>- Smartphone<br>- Laptop<br>- Other: [free text]	Displayed only if Q45 = Yes.
66	Conditional	During the past 2 weeks, what was the child's average viewing/use time per smart-device session? Enter numbers only, in hours.	Numeric field.	Displayed only if Q45 = Yes.
67	Conditional	During the past 2 weeks, what was the child's maximum viewing/use time per smart-device session? Enter numbers only, in hours.	Numeric field.	Displayed only if Q45 = Yes.
68	Conditional	During the past 2 weeks, what was the child's average daily smart-device use time, regardless of device type? Enter numbers only, in hours.	Numeric field.	Displayed only if Q45 = Yes.
69	Conditional	On average, how many days per week does the child use a smart device?	Numeric field.	Displayed only if Q45 = Yes.
70	Conditional	When the child uses a smart device, what is the distance between the device and the eyes?	Single choice:<br>- 50 cm or more (approximately the child's full arm length)<br>- 30-50 cm<br>- Within 30 cm (approximately forearm length)	Displayed only if Q45 = Yes.
