# Supplementary material for: Behavioral and Structural Correlates of Axial Length in School-Aged Children: Baseline Findings from the Seoul Myopia Cohort Study
Source: Life (Basel). 2026 Jul 16;16(7):1174. doi: 10.3390/life16071174 (PMC13412910; doi:10.3390/life16071174)
Supplement: Supplementary file 1 [file life-16-01174-s001.zip › Questionnaire_Original_Korean.pdf]

근시 환경요인 설문지

근시는 5~10세 경에 시작되어 20세 경에는 진행이 멈추는 경향이 있으나, 특히 초등학교 어린이에서 근시 진행속도가 빠른 편입니다. 본 설문을 통해 가족력과 안경이나 렌즈 착용 등 근시와 관련된 주변 환경을 평가하고 고도근시로의 진행 위험인자를 밝혀내고자 합니다. 본 설문에 성실히 참여해 주시는 학부모님에게 자제분 눈 건강과 관련된 상세한 검진 결과가 제공될 예정이오니 **꼭 참여해 주시기를 부탁드립니다**.

* 설문은 5분 정도 소요됩니다.

* 어머니의 답변이 필요한 문항이 많으므로 가급적 **대상자의 어머니**께서 답변해 주시기를 부탁 드립니다.

* 설문 결과는 **절대 공개되지 않으며,** 연구 목적으로만 사용되고 **연구가 종료되면 파기됩니다.**

* 표시는 필수 질문임

1. 이메일 *

2. **어린이 이름이 무엇인가요?** *

3. **현재 학년, 반, 번호**를 알려주세요. (예를 들어, 3학년 4반 12번인 경우 030412 로 적어 주시면 됩니다) *

4. **어린이 생년월일을 알려주세요** *

예: 2019년 1월 7일

5. **가장 최근 측정한 어린이 키는 얼마인가요? (cm단위, 숫자만 기입)** *

6. **가장 최근 측정한 어린이 몸무게는 얼마인가요? (kg단위, 숫자만 기입)** *

7. **어린이가 다니는 학교 이름이 무엇인가요?** *

한 개의 타원형만 표시합니다.

- 잠일 초등학교
- 잠신 초등학교
- 신천 초등학교
- 잠전 초등학교
- 석촌 초등학교

8. **어린이 성별을 알려주세요.** *

한 개의 타원형만 표시합니다.

- 남자
- 여자

9. **어린이가 몇 주에 태어났나요? (통상적인 재태기간은 40주입니다)(주 단위, 숫자만 기입)** *

10. **태어날 때 아이의 몸무게는 얼마였나요? (가능하면 소수점 첫째 자리까지 써주세요, 숫자만 기입)** *

11. **대상자 어린이의 출생 순서가 어떻게 되나요?** *

한 개의 타원형만 표시합니다.

- 첫째 아이
- 둘째 아이
- 셋째 이상
- 외동

12. **우리 아이는 쌍둥이다.** *

한 개의 타원형만 표시합니다.

- 예 *46번째 질문으로 건너뛰세요.*
- 아니오

13번째 질문으로 건너뛰세요.

제목 없는 섹션

13. **어린이를 출산했을 당시 아버지의 나이는 몇 세이셨나요? (만 나이, 숫자만)** *

____

14. **어린이를 출산했을 당시 어머니의 나이는 몇 세이셨나요? (만 나이, 숫자만)** *

____

15. **(어머니 대상) "임신 전" 혹은 "임신 중" 흡연을 하셨나요?** *

한 개의 타원형만 표시합니다.

- 아니오
- 예 *47번째 질문으로 건너뛰세요.*

16번째 질문으로 건너뛰세요.

제목 없는 섹션

16. **(어머니 대상) "임신 중" 음주를 하셨나요?** *

한 개의 타원형만 표시합니다.

- 아니오
- 예 *50번째 질문으로 건너뛰세요.*

17번째 질문으로 건너뛰세요.

제목 없는 섹션

17. **(어머니 대상) 임신 중 고혈압 (전자간증/임신중독증) 진단을 받으신 적이 있나요?** *

한 개의 타원형만 표시합니다.

- 아니오
- 예

18. **(어머니 대상) 임신 중 당뇨 진단을 받으신 적이 있나요?** *

한 개의 타원형만 표시합니다.

- 아니오
- 예

19. **(어머니 대상) 임신 중 상기 질환 이외 기타 질환을 받으신 적이 있나요? (있는 경우 기타란에 기술 부탁드립니다)** *

한 개의 타원형만 표시합니다.

- 아니오
- 기타:

20. **아이의 주된 수유방법은 어떤 것이었나요? (3개월까지)** *

한 개의 타원형만 표시합니다.

- 모유수유
- 분유
- 혼합 (모유, 분유)
- 기타: ____

21. **우리 아이는 출생 후 NICU (신생아중환자실) 치료를 받은 적이 있다.** *

한 개의 타원형만 표시합니다.

- 아니오
- 예

22. **우리 아이는 태아 때 호흡기질환을 진단받거나 산소 치료를 받은 적이 있다.** *

한 개의 타원형만 표시합니다.

- 아니오
- 예
- 모름

23. **우리 아이는 최근 1년 내에 정기 안과검진(시력검진)을 받은 적이 있다.** *

한 개의 타원형만 표시합니다.

- 아니오
- 예

24. **우리 아이는 미숙아망막병증 진단받거나, 치료를 받은 적이 있다.** *

한 개의 타원형만 표시합니다.

- 아니오
- 예

25. **우리 아이는 약시를 진단받은 적이 있다.** *

한 개의 타원형만 표시합니다.

- 아니오
- 예

26. **우리 아이는 사시를 진단받은 적이 있다.** *

한 개의 타원형만 표시합니다.

- 아니오
- 예

27. **이 밖에 기타 선천적인 안과질환을 진단받은 적이 있나요?** (있으면 기타란에 추가) *

한 개의 타원형만 표시합니다.

- 아니오
- 모름
- 기타:

28. **우리 아이는 말이나 행동발달이 느려서 발달 클리닉 진료를 본 적이 있다.** *

한 개의 타원형만 표시합니다.

- 아니오
- 예

29. **우리 아이는 발달 장애를 진단받은 적이 있다.** *

한 개의 타원형만 표시합니다.

- 아니오
- 예

30. **우리 아이는 현재 종합비타민(비타민 D 포함)을 복용하고 있다.** *

한 개의 타원형만 표시합니다.

- 아니오
- 예
- 모름

31. **지난 2주간 어린이의 평균 '수면 시간'은 얼마인가요? (시간 단위, 숫자만)** *

32. **지난 2주간 어린이의 평균 '취침 시작 시간'은 몇 시인가요? (24 시간 단위, 반올림하여 숫자만, 예를 들어 밤 11시면 "23")** *

33. **어린이의 성장 호르몬 치료를 받은 경험에 대해 알려주세요.** *

한 개의 타원형만 표시합니다.

- 현재 지속적으로 성장 호르몬 치료 중이다.
- 과거에 성장 호르몬 치료를 받은 적이 있으나 현재는 받고 있지 않다.
- 경험 없음.

34. **부모 중 근시가 있으면 알려주세요. (라식 등 굴절교정 수술을 받은 경우 '수술 전 상태' 기준)** *

한 개의 타원형만 표시합니다.

- 부
- 모
- 부모 둘 다 '근시'
- 부모 모두 '근시 없음'

35. **어린이의 형제자매 중 근시가 있으면 모두 알려주세요. (라식 등 굴절교정 수술을 받은 경우 그 전 상태를 알려주세요)** *

해당 사항에 모두 표시하세요.

- [ ] 외동이라 해당사항 없음
- [ ] 형/오빠
- [ ] 남동생
- [ ] 누나/언니
- [ ] 여동생
- [ ] 형제자매가 있지만 근시 없음

36. **우리 아이는 현재 근시가 있는 것으로 알고 있다.** *

한 개의 타원형만 표시합니다.

- 아니오
- 예

37. **우리 아이는 현재 '안경'을 일주일에 한 번 이상 착용한다** *

한 개의 타원형만 표시합니다.

- 아니오
- 예 *52번째 질문으로 건너뛰세요.*

38번째 질문으로 건너뛰세요.

제목 없는 섹션

38. **우리 아이는 현재 '드림렌즈'를 일주일에 한 번 이상 착용한다** *

한 개의 타원형만 표시합니다.

- 아니오
- 예 *56번째 질문으로 건너뛰세요.*

39번째 질문으로 건너뛰세요.

제목 없는 섹션

39. **우리 아이는 현재 '아트로핀 안약'을 일주일에 한 번 이상 점안한다** *

한 개의 타원형만 표시합니다.

- 아니오
- 예 *61번째 질문으로 건너뛰세요.*

40번째 질문으로 건너뛰세요.

제목 없는 섹션

40. **어린이의 하루 평균 "학교" 수업 시간은 얼마인가요? (방학 제외, 하루 평균 시간)** *

41. **어린이의 하루 평균 "학원 (학습학원, 학습지 포함)" 수업 시간은 얼마인가요? (방학 포함, 하루 평균 시간)** *

42. **어린이의 하루 평균 독서/숙제 시간은 얼마인가요? (방학 포함, 하루 평균 시간)** *

43. **어린이의 하루 평균 *야외 활동 시간은 얼마인가요? (주말 포함, 시간 단위, 숫자만)** *
(* 등하교를 포함해 실외에서 하는 모든 활동을 의미합니다)

44. **1달에 자연체험**(산책, 피크닉, 캠핑, 숲체험 등)**을 하는 평균 횟수는 얼마나 되나요? (1달 동안 평균 일수)** *

45. **어린이가 스마트기기(태블릿, 스마트폰, 노트북)를 일주일에 1회 이상 시청하나요?** *

한 개의 타원형만 표시합니다.

- ◯ 아니오
- ◯ 예 *65번째 질문으로 건너뛰세요.*

쌍둥이

46. 어린이가 일란성 혹은 이란성 쌍둥이인지 알려주세요

한 개의 타원형만 표시합니다.

- 일란성 *13번째 질문으로 건너뛰세요.*
- 이란성 *13번째 질문으로 건너뛰세요.*

13번째 질문으로 건너뛰세요.

흡연

47. (어머니 대상) **임신 전 하루 평균 흡연량은 어떻게 되시나요? (평균 개비 수/일)**

____

48. (어머니 대상) **임신 전 총 흡연 기간은 어떻게 되시나요? (반올림하여 1년 단위로)**

____

49. (어머니 대상) **언제 끊으셨나요?**

한 개의 타원형만 표시합니다.

- 임신 전 *16번째 질문으로 건너뛰세요.*
- 임신 직후 - 임신 12주 *16번째 질문으로 건너뛰세요.*
- 임신 13주 -46주 *16번째 질문으로 건너뛰세요.*
- 임신 46주 이후 *16번째 질문으로 건너뛰세요.*
- 끊지 않았음 *16번째 질문으로 건너뛰세요.*

16번째 질문으로 건너뛰세요.

음주

50. **(어머니 대상) 임신 중 평균 음주 횟수는 어떻게 되나요? (일주일에 몇 회, 숫자만)**

____

51. **(어머니 대상) 임신 중 평균 음주량은 어떻게 되나요? (술자리당 평균 잔 수, 종류 불문, (예시) 소주 1병=8잔, 맥주 1병=3잔)**

____

17번째 질문으로 건너뛰세요.

안경

52. **우리 아이가 안경 착용을 시작한 만 나이는?**

____

53. **최근 2주간 하루 평균 안경착용 시간은 어떻게 되나요? (1시간 단위, 숫자만)**

____

54. **안경착용은 1주일 동안 평균적으로 몇 일 하나요? (일 단위, 숫자만)**

____

55. **현재 착용하는 안경을 처방받은지 얼마나 시간이 지났나요? (1달 단위, 숫자만)**

____

38번째 질문으로 건너뛰세요.

드림렌즈

56. **우리 아이가 드림렌즈 착용을 시작한 만 나이는?**

____

57. **드림렌즈 착용 부위는?**

한 개의 타원형만 표시합니다.

- 양쪽 눈 모두
- 오른쪽 눈만 착용
- 왼쪽 눈만 착용

58. **최근 2주간 하루 평균 드림렌즈 착용 시간은 어떻게 되나요? (1시간 단위, 숫자만)**

____

59. **드림렌즈는 1주일 동안 평균 몇 회 착용하나요? (일 단위, 숫자만)**

____

60. **현재 착용하는 드림렌즈를 처방받은지 얼마나 시간이 지났나요? (1달 단위, 숫자로)**

____

39번째 질문으로 건너뛰세요.

아트로핀

61. **우리 아이가 아트로핀 안약 사용을 시작한 만 나이는?**

____

62. **아트로핀 점안 부위는?**

한 개의 타원형만 표시합니다.

- 양쪽눈 모두
- 오른쪽 눈
- 왼쪽 눈

63. **사용중인 아트로핀 농도는?**

한 개의 타원형만 표시합니다.

- 0.1%
- 0.05%
- 0.025%
- 0.01%
- 모름

64. **아트로핀 안약은 1주일에 평균 몇 일 점안하나요? (하루 단위, 숫자만)**

____

40번째 질문으로 건너뛰세요.

스마트기기

65. **지난 2주간 제일 사용빈도가 높은 스마트기기는 무엇인가요?**

한 개의 타원형만 표시합니다.

- 태블릿 (아이패드, 갤럭시탭)
- 스마트폰
- 노트북
- 기타: ____

66. **지난 2주간 스마트기기 사용시 1회당 평균 시청 시간은? (시간 단위, 숫자만)**

____

67. **지난 2주간 스마트기기 사용시 1회당 최대 시청 시간은? (시간 단위, 숫자만)**

68. **지난 2주간 하루 평균 스마트기기 (종류 불문) 사용 시간은? (시간 단위, 숫자만)**

69. **스마트기기를 1주일 동안 평균적으로 몇 일 사용하나요? (일 단위, 숫자만)**

70. **스마트기기 시청시, 기기와 눈 사이 거리는?**

한 개의 타원형만 표시합니다.

- 50cm (어린이 전체 팔 길이) 이상
- 30cm 이상 50cm 이하
- 30cm (앞팔 길이) 이내

이 콘텐츠는 Google이 만들거나 승인하지 않았습니다.

Google 설문지
