# Supplementary material for: Behavioral and Structural Correlates of Axial Length in School-Aged Children: Baseline Findings from the Seoul Myopia Cohort Study
Source: Life (Basel). 2026 Jul 16;16(7):1174. doi: 10.3390/life16071174 (PMC13412910; doi:10.3390/life16071174)
Supplement: Supplementary file 1 [file life-16-01174-s001.zip › Table S1.pdf]

Supplementary Table S1. Unadjusted associations between candidate risk factors and axial length in the study population

**Variables**	**Beta**	**95% CI**	***P* value**
Demographic and family factors			
Age	0.278	(0.218, 0.338)	<0.001
Sex			
Boys	Reference		
Girls	-0.415	(-0.602, -0.228)	<0.001
Birth order	0.109	(-0.022, 0.240)	0.104
Twin status			
Singleton	Reference		
Twin	-0.328	(-0.773, 0.118)	0.149
Paternal age at birth	0.008	(-0.032, 0.015)	0.485
Maternal age at birth	0.001	(-0.024, 0.025)	0.964
Number of myopic parents	0.186	(0.074, 0.297)	0.001
Sibling myopia	0.097	(-0.071, 0.265)	0.259
Anthropometric and Perinatal factors			
Height	0.043	(0.034, 0.051)	<0.001
Weight	0.035	(0.024, 0.046)	<0.001
Body mass index	0.033	(0.000, 0.066)	0.051
Gestational age	0.014	(-0.043, 0.071)	0.638
Birth weight	0.215	(0.017, 0.413)	0.034
Maternal comorbidities	-0.061	(-0.298, 0.176)	0.613
Gestational diabetes	0.184	(-0.208, 0.577)	0.356
Thyroid disorders	0.317	(-0.361, 0.994)	0.359
Smoking	0.466	(-0.173, 1.105)	0.153
NICU admission			
No			
Yes	0.280	(-0.136, 0.696)	0.186
Neonatal oxygen therapy			
No			
Yes	0.613	(-0.063, 1.288)	0.076
Feeding method	-0.315	(-0.611, -0.019)	0.037
Breast Feeding	Reference		
Formula Feeding	-0.315	(-0.611, -0.019)	0.037
Developmental delay			
No	Reference		
Yes	-0.062	(-1.409, 1.285)	0.928
Lifestyle and Environmental Factors			
Smart device usage pattern			
Current device usage (Y*es vs. No*)	0.410	(-0.022, 0.843)	0.063
Primary device type			

Non-user	Reference		
Smartphone usage	0.544	(0.093, 0.996)	0.018
Tablet PC usage	0.323	(-0.119, 0.765)	0.152
TV/laptop usage	0.413	(-0.119, 0.945)	0.128
Usage duration & frequency			
Ave daily screen time	0.024	(-0.053, 0.102)	0.536
Max daily screen time	0.067	(-0.004, 0.139)	0.066
Ave duration per session	0.140	(0.035, 0.246)	0.009
Weekly frequency	0.031	(-0.010, 0.072)	0.140
Viewing distance			
Non-user	Reference		
<30 cm	0.579	(0.123, 1.036)	0.013
30–50 cm	0.318	(-0.124, 0.759)	0.158
>50 cm	0.443	(-0.058, 0.945)	0.083
School time	0.257	(0.146, 0.369)	<0.001
After school academy time	0.082	(0.009, 0.155)	0.028
Homework time	0.108	(-0.010, 0.226)	0.072
Outdoor activity duration	0.050	(-0.019, 0.119)	0.157
Outdoor activity frequency	0.010	(-0.031, 0.050)	0.640
Average sleep duration	-0.177	(-0.309, -0.046)	0.008
