# Supplementary material for: Behavioral and Structural Correlates of Axial Length in School-Aged Children: Baseline Findings from the Seoul Myopia Cohort Study
Source: Life (Basel). 2026 Jul 16;16(7):1174. doi: 10.3390/life16071174 (PMC13412910; doi:10.3390/life16071174)
Supplement: Supplementary file 1 [file life-16-01174-s001.zip › Table S2.pdf]

Supplementary Table S2. Absolute macular OCT parameters according to grade

Variable	Grade 1 (n=182)	Grade 2 (n=182)	Grade 3 (n=158)	Grade 4 (n=146)	Grade 5 (n=112)	Total (n=775)	*P* value†
Fovea center, µm	237.5 ± 15.0	236.6 ± 18.1	243.0 ± 16.8	246.4 ± 19.0	249.2 ± 16.0	241.8 ± 17.6	<0.001
Inner superior, µm	318.6 ± 15.7	316.9 ± 15.1	321.5 ± 14.4	321.1 ± 13.7	326.0 ± 13.4	320.3 ± 14.9	<0.001
Inner temporal, µm	306.3 ± 14.6	304.8 ± 16.1	309.7 ± 13.5	308.1 ± 14.6	313.2 ± 13.3	308.0 ± 14.8	<0.001
Inner inferior, µm	314.7 ± 14.7	311.6 ± 16.0	316.0 ± 14.0	314.7 ± 14.2	320.3 ± 13.0	315.1 ± 14.8	<0.001
Inner nasal, µm	319.9 ± 15.0	318.0 ± 16.0	320.8 ± 14.8	320.9 ± 15.0	326.6 ± 13.9	320.8 ± 15.2	<0.001
Outer superior, µm	288.5 ± 15.0	282.1 ± 13.6	284.3 ± 13.2	282.5 ± 11.3	286.7 ± 13.1	284.8 ± 13.6	<0.001
Outer temporal, µm	267.2 ± 13.9	262.5 ± 13.4	265.8 ± 11.8	262.3 ± 12.2	267.4 ± 12.2	264.9 ± 13.0	<0.001
Outer inferior, µm	274.1 ± 14.9	269.2 ± 14.0	269.0 ± 12.4	266.2 ± 12.9	272.3 ± 12.5	270.2 ± 13.8	<0.001
Outer nasal, µm	303.7 ± 16.4	298.5 ± 15.1	299.9 ± 13.7	297.5 ± 13.2	303.5 ± 13.5	300.5 ± 14.8	<0.001
Choroidal thickness, µm	257.0 ± 38.1	251.7 ± 39.5	252.8 ± 44.8	232.8 ± 53.0	251.0 ± 43.1	249.5 ± 44.3	<0.001
Average thickness, µm	282.7 ± 13.5	278.0 ± 12.8	280.9 ± 11.4	278.7 ± 11.4	284.1 ± 10.5	280.7 ± 12.3	<0.001
Macular volume, mm^3	10.18 ± 0.49	10.01 ± 0.46	10.11 ± 0.41	10.03 ± 0.41	10.23 ± 0.38	10.10 ± 0.44	<0.001

Abbreviations: OCT, optical coherence tomography; SD, standard deviation.
Data are expressed as mean (standard deviation) and summarized on a per-eye basis.
The ETDRS macular grid was defined as follows: the central foveal subfield corresponded to the central 1-mm circle, the inner sectors to the 1- to 3-mm ring, and the outer sectors to the 3- to 6-mm ring.
† P values were calculated using one-way analysis of variance across grades.
