# Supplementary material for: Behavioral and Structural Correlates of Axial Length in School-Aged Children: Baseline Findings from the Seoul Myopia Cohort Study
Source: Life (Basel). 2026 Jul 16;16(7):1174. doi: 10.3390/life16071174 (PMC13412910; doi:10.3390/life16071174)
Supplement: Supplementary file 1 [file life-16-01174-s001.zip › Table S3.pdf]

Supplementary Table S3. Multivariable associations of chronological age with optical coherence tomography parameters, adjusted for axial length (AL, Model 1) or spherical equivalent (SE, Model 2).

**Variable**	**Model 1: adjusted for AL**			**Model 2: adjusted for SE**		
	**beta**	**95% CI**	***P* value**	**beta**	**95% CI**	***P* value**
Fovea center, µm	2.24	(0.28, 4.20)	0.025	2.32	(0.39, 4.25)	0.019
Inner superior, µm	1.33	(-0.29, 2.95)	0.109	0.86	(-0.72, 2.44)	0.284
Inner temporal, µm	1.25	(-0.37, 2.87)	0.130	0.84	(-0.74, 2.42)	0.296
Inner inferior, µm	1.37	(-0.44, 3.18)	0.137	0.74	(-1.02, 2.50)	0.409
Inner nasal, µm	1.16	(-0.53, 2.85)	0.179	0.66	(-0.99, 2.30)	0.433
Outer superior, µm	-0.21	(-1.64, 1.23)	0.776	-0.93	(-2.36, 0.50)	0.202
Outer temporal, µm	0.35	(1.00, 1.71)	0.606	-0.34	(-1.69, 1.00)	0.614
Outer inferior, µm	-0.03	(-1.45, 1.38)	0.963	-0.74	(-2.16, 0.68)	0.304
Outer nasal, µm	0.36	(-1.27, 1.98)	0.668	-0.28	(-1.89, 1.33)	0.731
Average thickness, µm	0.46	(-0.86, 1.78)	0.495	-0.05	(-1.35, 1.26)	0.945
Macular volume, mm^3	0.02	(-0.03, 0.06)	0.500	-0.00	(-0.05, 0.04)	0.939
Choroidal thickness, µm	1.53	(-3.15, 6.21)	0.521	-1.03	(-5.61, 3.56)	0.660

Abbreviations: CI, confidence interval

The ETDRS macular grid was defined as follows: the central foveal subfield corresponded to the central 1-mm circle, the inner sectors to the 1- to 3-mm ring, and the outer sectors to the 3- to 6-mm ring.
